# Glutathione Metabolism and the Novel Role of Mitochondrial GSH in Retinal Degeneration

**DOI:** 10.3390/antiox10050661

**Published:** 2021-04-24

**Authors:** Parameswaran G. Sreekumar, Deborah A. Ferrington, Ram Kannan

**Affiliations:** 1 The Stephen J. Ryan Initiative for Macular Research (RIMR), Doheny Eye Institute, Los Angeles, CA 90033, USA; sparameswaran@doheny.org; 2 Department of Ophthalmology and Visual Neurosciences and Stem Cell Institute, University of Minnesota, Minneapolis, MN 55455, USA; ferri013@umn.edu; 3 Stein Eye Institute, Geffen School of Medicine, University of California, Los Angeles, CA 90095, USA

**Keywords:** retinal degeneration, mitochondrial GSH, RPE, SLC25A10 (DIC), SLC25A11 (OGC), bioenergetics

## Abstract

Glutathione (GSH) is present ubiquitously, and its role as a crucial cellular antioxidant in tissues, including the retina, is well established. GSH’s antioxidant function arises from its ability to scavenge reactive oxygen species or to serve as an essential cofactor for GSH S-transferases and peroxidases. This review summarizes the general functions, retinal distribution, disorders linked to GSH deficiency, and the emerging role for mitochondrial GSH (mGSH) in retinal function. Though synthesized only in the cytosol, the presence of GSH in multiple cell organelles suggests the requirement for its active transport across organellar membranes. The localization and distribution of 2-oxoglutarate carrier (OGC) and dicarboxylate carrier (DIC), two recently characterized mitochondrial carrier proteins in RPE and retina, show that these transporters are highly expressed in human retinal pigment epithelium (RPE) cells and retinal layers, and their expression increases with RPE polarity in cultured cells. Depletion of mGSH levels via inhibition of the two transporters resulted in reduced mitochondrial bioenergetic parameters (basal respiration, ATP production, maximal respiration, and spare respiratory capacity) and increased RPE cell death. These results begin to reveal a critical role for mGSH in maintaining RPE bioenergetics and cell health. Thus, augmentation of mGSH pool under GSH-deficient conditions may be a valuable tool in treating retinal disorders, such as age-related macular degeneration and optic neuropathies, whose pathologies have been associated with mitochondrial dysfunction.

## 1. Background

Glutathione (GSH), one of the most abundant non-protein thiols present at millimolar concentrations in mammalian tissues, is involved in a plethora of functions stemming from combating oxidative stress to immune function and fibrogenesis [1,2,3,4]. GSH exists in reduced and disulfide-oxidized (GSSG) forms [5]. Under physiological conditions, the reduced GSH is the major form and accounts for >98% of total GSH [5,6]. About 80–85% of the cellular GSH is present in the cytosol, and 10–15% is associated with mitochondria, with a small fraction found in the endoplasmic reticulum (ER) and nucleus [7,8,9,10]. In most of these compartments, GSH is typically found in a highly reduced state, but, in the ER, a substantial portion is oxidized, whereas in the cytoplasm the oxidized form is usually on the order of about 1% of the total or less [10,11,12].

**Figure 1:**
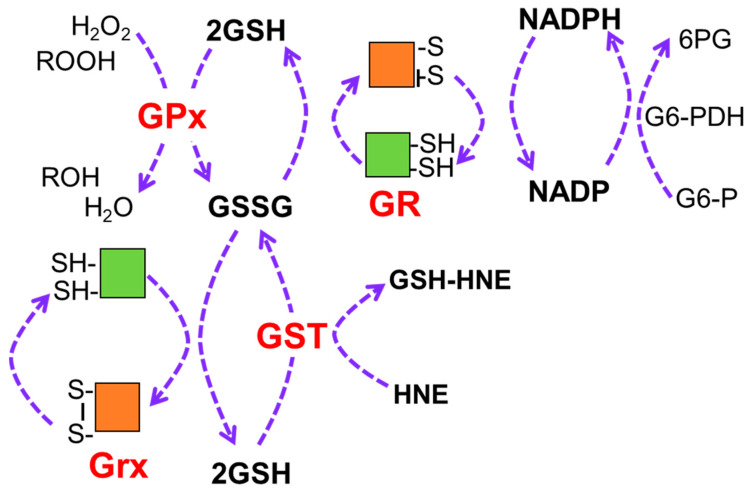
Redox cycling regenerates antioxidants and requires GSH. Antioxidants undergo multiple rounds of reduction–oxidation at active site cysteine residues, which are represented by the disulfide (S-S) and SH, respectively. GSH provides the reducing equivalents for these reactions involving the reduction in harmful hydrogen peroxide and organic peroxides (ROOH) by GPx or neutralization of reactive aldehydes, such as HNE, through addition of GSH by GST. GSSG is replenished to GSH by either NADP-dependent GR or Grx. NADPH is produced by reaction with glucose 6-phosphate dehydrogenase. NADPH, nicotinamide adenine dinucleotide phosphate; GSH, reduced glutathione; GSSR, oxidized glutathione; GR, glutathione reductase; GPx, glutathione peroxidase; Grx, glutaredoxin; GST, glutathione S-transferase; G6-PDH, glucose 6-phosphate dehydrogenase.

The requirement for large quantities of GSH is likely due to its role in multiple processes, including protecting proteins during an oxidative stress through the reversible glutathionylation of active thiols. GSH also maintains the reduced form of multiple antioxidant enzymes through the process of redox cycling, which involves repeated reduction–oxidation (SH to SS) reactions at active site cysteine residues (Figure 1). The main role for this antioxidant system is to eliminate harmful peroxides, such as hydrogen peroxide and organic peroxides, via their reduction by GSH peroxidase (GPx), with GSH serving as a cofactor. The oxidized form (GSSG) is predominantly produced by the catalysis of GSH peroxidase (GPx), as well as from the direct reactions of GSH with electrophilic compounds and reactive aldehydes, such as 4-hydroxy-2-nonenal (HNE), catalyzed by GSH S-transferase (GST). GSSG can be recycled back to GSH by either nicotinamide adenine dinucleotide phosphate (NADPH)-dependent GSH reductase (GR) or glutaredoxin (GRX) such that the GSH pool is markedly reduced with low levels of GSSG being present [13,14]. Thus, quantifying the ratio of GSSG to GSH is considered as one of the best indicators of cellular oxidative stress.

## 2. Glutathione Biosynthesis

**Figure 2:**
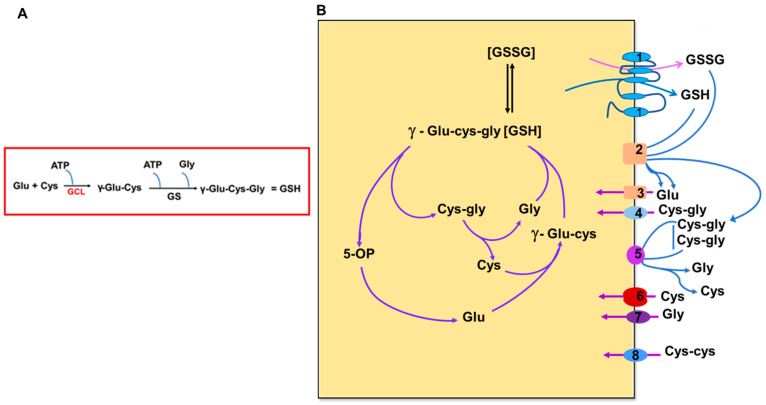
Scheme showing the biosynthesis of GSH (**A**), and the glutathione cycle and transport of constituent amino acids and related substrates (**B**). Figure 2B is modified from Bachhawat AK, Yadav S. The glutathione cycle: glutathione metabolism beyond the γ-glutamyl cycle. IUBMB Life. 2018 July; 70(7):585–592. doi: 10.1002/iub.1756. Epub 2018 Apr 17. PMID: 29667297. Wiley Publishers [19]. GCL—glutamate–cysteine ligase, GS—glutathione synthetase, GSH—glutathione. 5-OP-5-oxoproline. Numbers refer to representative transporters involved. 1–2. MRP family; 3. EAATS family; 4. Peptide transporters; 5–6. System Xc^−^ and other transporters; 7. Glycine transporter; 8. Disulfide transporter.

GSH biosynthesis is a multi-step process in which the three precursor amino acids, cysteine, glutamate, and glycine, are combined to form the tripeptide GSH (Figure 2A). GSH is synthesized exclusively in the cytosol by the sequential action of two ATP-dependent enzymes, glutamate–cysteine ligase (GCL) and glutathione synthetase (GS) [15]. The GCL enzyme is composed of a catalytic unit (GCLC) and a modifier subunit (GCLM), which are encoded by different genes [14]. The catalytic unit contains all substrate binding sites, whereas the regulatory unit modulates the affinity of the active subunit for substrates and inhibitors [14]. Genetic elimination of each subunit provides some indication of their relative importance and contribution to cell protection. Mice lacking GCLM demonstrate no outward phenotype but exhibit a marked decrease in GSH and increased sensitivity to toxic insults [16]. Mice lacking GCLC die before birth [17]. GCL catalyzes the first, rate-limiting step of the biosynthetic pathway by which glutamate and cysteine are linked to produce γ-glutamylcysteine in the presence of ATP and Mg2+ or Mn2+ [18]. The last step of the biosynthetic pathway of GSH synthesis is by the addition of glycine to the γ-glutamylcysteine intermediate catalyzed by GSH synthetase (Figure 2) [14]. GSH can be hydrolyzed to cysteinyl glycine and 5-oxoproline by γ-glutamyl cyclotransferase activity of cation transport regulator-like protein 1 (CHAC1) [19,20]. Glutamate is formed by the breakdown of 5-oxoproline in the presence of 5-oxoprolinase, while cysteinyl glycine is cleaved by respective peptidases to yield cysteine and glycine. These newly liberated amino acids can be reused for the synthesis of GSH [19].

## 3. Glutathione Function in Cells and Tissues

As discussed previously, GSH is required for the maintenance and regulation of tissue redox homeostasis. Its multi-faceted functions include contributions to key pathways, such as the regulation of transcription factors involved in redox signaling, metabolism of estrogens, leukotrienes, and prostaglandins, cellular proliferation, apoptosis, and the detoxification of many endogenous compounds and xenobiotics [5,10]. A deficiency of GSH or a major change in the glutathione/glutathione disulfide (GSH/GSSG) ratio renders cells or cellular organelles vulnerable to stress-induced damage. The resulting tissue injury is believed to be associated with the induction and or progression of several neurodegenerative diseases, autoimmune diseases and ocular disorders, such as age-related macular degeneration (AMD), glaucoma, Leber’s Hereditary Optic Neuropathy, and diabetic retinopathy [21,22,23,24,25,26,27]. The decline in retinal GSH levels in the above conditions is likely dictated by factors, such as the severity of stress and disease, genetics, age, gender, or environmental factors.

**Figure 3:**
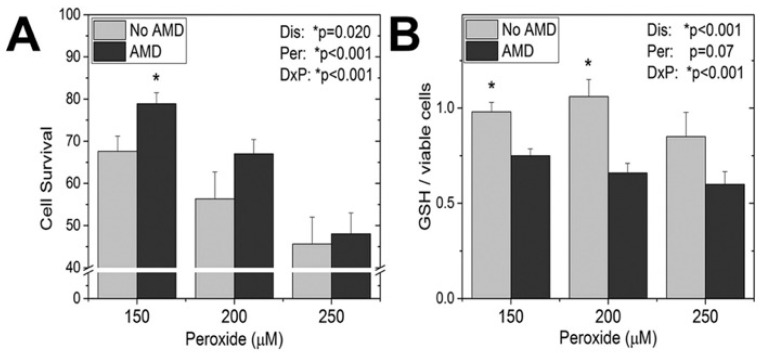
Effect of oxidative stress on cell survival (**A**) and GSH levels (**B**) in RPE isolated from AMD donors and age-matched controls (no AMD). Results were measured 24 h after RPEs were exposed to different doses of hydrogen peroxide. Modified from Redox Biol. 2017; 13:255–265, Ferrington et al. [21]. Copyright (2021), with permission obtained from Elsevier. * *p* < 0.05 as determined by 2-way ANOVA and Tukey’s post-hoc test. Results from 2-way ANOVA for disease (Dis), peroxide dose (Per), and their interaction (DxP) are shown on the graphs.

In contrast to the neurodegeneration and lower basal GSH levels observed at late stages of disease, there is evidence that cells experiencing earlier disease have increased protection from oxidative stress through utilization of GSH. For example, cell protection from hydrogen peroxide, which is directly detoxified by GSH or other antioxidants involved in redox cycling (Figure 3), was observed in primary RPE cultures from AMD donors [21]. It is important to note that these donors were at early stages of AMD, prior to vision loss. While a dose-dependent decrease in cell survival was observed in RPE from donors with and without AMD, the RPE from AMD donors were more resistant to peroxide-induced death (Figure 3A). Additional experiments that reported RPE from AMD donors were also more resistant to peroxide-induced reductions in both mitochondrial and glycolytic function. The resistance to oxidative damage by AMD RPE was attributed to their increased utilization of GSH after exposure to peroxide. This idea is supported by measurement of GSH content after exposure to increasing doses of peroxide, which showed that GSH depletion was significantly greater in cells from AMD donors (Figure 3B). While the mechanism responsible for the coordinate reduction in GSH and resistance to cell death in AMD RPE donors was not explored, GSH protection could occur by eliminating peroxide via by GSH peroxidase (Figure 1) or through the reversible glutathionylation of critical cysteine residues. These results provide additional evidence of the importance of GSH in protecting the cell from oxidative damage and cell death.

A link between deficiency of GSH and pathological changes associated with epithelial–mesenchymal transition (EMT) has been shown mainly in studies with cultured cells [28] EMT process has been demonstrated to play a pivotal role in posterior capsular opacification, a postoperative complication of cataract surgery that occurs due to the proliferation, migration, and transformation of remnant lens epithelial cells [29]. Data from the lens-conditional gamma glutamyl-cysteine ligase subunit (GCLC) knockout (KO) mice (LEGSKO mice) showed the deficiency of GSH promoted EMT via the regulation of the Wnt/catenin pathway in lens epithelial cells [30]. EMT has been reported in intraocular fibrotic disorders, such as proliferative vitreoretinopathy [31,32], where migration of RPE into the vitreous has been observed. Given the widely accepted fact that GSH is involved in several ocular diseases, there is considerable interest in formulating therapies focused on regulating GSH levels, especially mitochondrial GSH (mGSH), which could help to modulate disease risk or progression.

## 4. GSH Distribution in the Retina and RPE in Health and Disease

The retina has one of the highest oxidative metabolic rates per tissue weight [33,34]. Increased oxygen flux, continuous exposure to light, and the availability of easily oxidized polyunsaturated fatty acids promote an environment in the retina that is highly susceptible to oxidation. Therefore, an effective antioxidant system is required to protect retinal tissues from the continuous exposure to reactive oxygen species. The presence of the GSH antioxidant system, including the enzymes involved in GSH metabolism and regeneration (Figure 1), has been well documented in retinal cells, such as photoreceptor outer segments, Müller glial cells, RPE cells, retinal astrocytes [1,35,36,37,38,39]. Data from gene and protein expression are consistent with GSH as one of the most prominent antioxidants in retina and RPE cells [38,40].

GSH distribution and content demonstrates cell-type-dependent, as well as stress-dependent, properties. In a study examining the effect of ischemia on the cellular distribution of GSH in the rat retina, a redistribution of GSH from Müller glia and astrocytes to neuronal cells was demonstrated by the gradual increase in staining [41]. In normal monkey retina, GSH labeling was found primarily confined to Müller cells and horizontal cell bodies through retinal neurons. However, in the glaucomatous retinas, Müller cell immunoreactivity for GSH was always greater [42]. Immunogold labeling studies in adult pig retina showed the strongest immunolabeling for GSH in the RPE cells and in choroid fibroblasts. Intermediate densities of gold particles were recorded in Müller cells and photoreceptor inner segments [40]. However, subcellular localization revealed immunoreactivity was enriched in the mitochondria relative to the cytoplasmic matrix [40]. Intraperitoneal injection of l-buthionine sulfoximine (BSO), an irreversible inhibitor of GCL, in mice caused GSH depletion and increased cell death. BSO treatment first affected the cells of the inner nuclear layer before cells in other layers of the retina [43]. This was attributed to the oxidation of GSH in some layers and the activation of GSH synthesis in other retinal layers. While the diseases associated with the deficiency of endogenous antioxidants are far too many to list, information on the link between GSH and retinal diseases for select publications directly relevant to this review is presented in Table 1.

## 5. Cellular Plasma Membrane Transport of GSH

The multiple functions of GSH emphasize the absolute necessity for maintaining adequate GSH pools throughout the cell and its organelles. Considering the properties of GSH, such as the size (307 Da) and net negative charge, GSH transport requires carrier proteins to cross membranes to reach its many targets. Thus, the two main mechanisms for maintaining GSH pools in mammalian cells is GSH uptake via specific transporters and the other through the uptake of amino acids for de novo synthesis of GSH (Figure 2) [5,68,69]. The transport of GSH across the plasma membrane is controlled by a switch mechanism of the open/closed configuration of the GSH transporters. This transport is uniport and cells generally efflux GSH rather than import as cellular GSH levels are higher than in the intracellular medium. These GSH transporters in the plasma membrane have been known for many decades, and include early reports on the biochemical characterization of GSH transport across the plasma membrane [69,70,71].

The first identified GSH transporter was the multidrug resistance-associated proteins (MRP), a sub-class of the ATP-Binding Cassette (ABC) transporter superfamily. The MRP family of transporters are found on the plasma membranes of many cells including RPE cells [1]. Furthermore, eight other members of this family (MRP2-9) have been discovered, each with evidence to support GSH conjugates, and other GSH species, as substrates [5]. The MRP transporters are demonstrated as cotransporters of organic anions (OA−) and GSH [5,72], GSH-conjugated xenobiotics (GS-XN), and GSH-conjugated metabolites. This efflux offers drug resistance to tumor cells and can protect normal cells from toxic insults. MRP1 functions as a GSH-conjugate transporter not only at the plasma membrane but also in intracellular secretory vesicles [73]. Screening of GSH/GSSG efflux transporters revealed MRP1, MRP2, MRP3, MRP4, MRP5, MRP6, and MRP7 are present at the transcript level in the RPE cells among which MRP1 was the most abundant [1]. MRP1 was localized to the plasma membrane, and inhibition of MRP1 markedly decreased GSH efflux [1]. GSH efflux was significantly higher in MRP1-overexpressing RPE cells, which also contained lower levels of cellular GSH and GSSG [1]. However, these efflux pumps had broad substrate specificity and low affinity for GSH and, despite displaying the capacity for GSH transport, appeared to be primarily required for the efflux of GSH conjugates (for example, nitrolinoleic acid), rather than GSH [5].

## 6. Mitochondrial GSH and Its Critical Role

Although they lack the synthetic machinery, multiple organelles, including the nucleus, endoplasmic reticulum, and mitochondria, have their own GSH pool, with reduced GSH to oxidized GSSG that vary between the organelle [74]. In the mitochondria, GSH mainly occurs in a reduced form [75]. This GSH pool allows the mitochondria to resist oxidant insults and to neutralize the superoxide that is generated during the production of ATP as a result of the direct transfer of electrons to molecular oxygen [76,77]. The presence of an effective antioxidant system led by GSH is especially critical when considering that the steady state concentration of superoxide is estimated to be 5–10 fold greater in the mitochondrial matrix than in the cytosol [76].

Emerging studies indicate a pivotal role of mitochondria in initiating multiple signals in response to metabolic and genetic stress that affects nuclear gene expression, causing changes in cell function [77]. mGSH plays a crucial role in the mitochondria, some of which includes its role as an antioxidant, as a detoxifying agent of xenobiotics, a stabilizer of mitochondrial DNA, as a cofactor for Fe-S cluster synthesis [14]. GSH is also a redox regulator of electron transport chain (ETC) proteins that perform oxidative phosphorylation, a process involving the sequential transfer of electrons between the five ETC protein complexes embedded in the inner mitochondrial membrane (IMM) [78,79]. Regulation of these ETC proteins suggest a possible link between mitochondrial metabolism and redox homeostasis through mGSH status [80]. mGSH depletion significantly decreased mitochondrial basal respiration and ATP production, and the reserve capacity in human RPE cells suggests an oxidative stress-dependent mechanism [81]. Supporting findings have been reported in previous studies with other cell types showing the dependence of mitochondrial function and respiration on mGSH levels [82]. ETC protein expression data suggested that ETC complex II was one of the main target sites where 2-oxoglutarate carrier (OGC) and dicarboxylate carrier (DIC) inhibitors (see below) executed their potential inhibitory effect on the respiratory chain. Treatment of RPE cells with phenyl succinate (PS—an inhibitor of OGC) or butylmalonate (BM—an inhibitor of DIC) caused a disruption of complex II. It has been demonstrated that acute oxidative stress to the mitochondria caused significant vulnerability to complex I in rat hepatocytes [80]. However, no significant change was noticed in the respiratory chain complex V.

## 7. Mitochondrial Import and Export of GSH

As mentioned earlier, mitochondria are extremely sensitive to the damaging effects of free radicals. Therefore, these organelles are enriched with an array of free radical scavenging systems. Of specific note, the mitochondrial GSH pool is a critical antioxidant reserve that is transported entirely from the cytosolic pool via facilitated transport. As GSH has a net negative charge at physiological pH, the high concentration of mGSH suggests the presence of specific transport systems that work against an electrochemical gradient [83,84,85]. The concentration of GSH in mitochondria is similar to that of cytosol (10–14 mM). As ubiquitous, semi-autonomous cellular organelles, mitochondria are separated from the cytoplasm by a double membrane, the outer and IMM. Therefore, the membranes of the mitochondria must harbor transporters or channels that facilitate GSH transport. The mechanism of GSH transport across the outer membrane (OMM) appears to be relatively uncomplicated because the porins allow molecules smaller than ~5 kDa to diffuse from the intermembrane space (IMS) across the outer mitochondrial membrane to the cytosol, including small proteins and GSH. However, further evidence is needed to determine whether this is the case for both GSH and GSSG [86]. Moreover, although GSH can cross the OMM, its transport into the mitochondrial matrix cannot be explained by simple diffusion. Of note, the IMM and OMM of the mitochondrion differ strongly with respect to the protein and lipid composition [75,87] and permeability as the IMM is impermeable to several solutes and molecules, including GSH. Therefore, mGSH is imported from the cytosolic by the activity of specific carriers [83]. While the transport of mGSH is not fully understood, evidence from reconstitution assays in proteoliposomes testing for substrate specificity, kinetics, dependence on membrane potential, and sensitivity to carrier-selective inhibitors identified two potential members of the mitochondrial carrier family (SLC25), the mitochondrial dicarboxylate carrier (DIC; SLC25A10) and the 2-oxoglutarate carrier (OGC; SLC25A11) [88,89,90,91,92] as mitochondrial GSH transporters. OGC imports cytosolic GSH into mitochondria in exchange for 2-oxoglutarate (2-OGC) and other dicarboxylates (usually malate). DIC mediates electroneutral exchange of dicarboxylates or GSH for inorganic phosphate [93,94]. OGC was shown to have overlapping substrate specificities with some other mitochondrial carriers, but none of these carriers shared the complete group of OGC substrates (2-oxoglutarate, oxaloacetate, malonate, malate). The citrate carrier (CIC) overlaps with OGC by transporting the substrates malate and malonate [95]. The role of OGC as the porphyrin transporter, which is required for the mitochondrial import of the precursor porphyrin for final conversion to heme, has also been reported [96].

**Figure 4:**
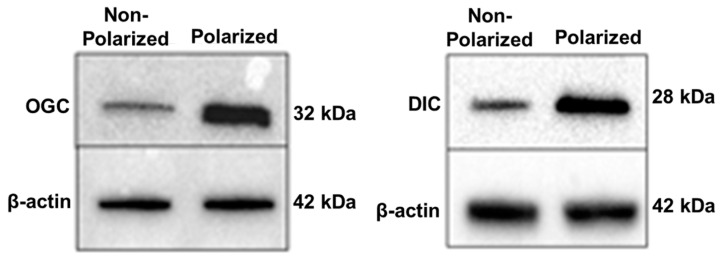
Polarity upregulates mitochondrial carrier proteins, OGC and DIC, in primary human RPE cells. Polarized RPE cultures had an average TER of 380 ± 60 Ω·cm^2^ (reproduced from [97] and is licensed under a Creative Commons Attribution-NonCommercial-NoDerivatives 4.0 International License).

The Kannan laboratory was the first to report the expression, localization, and the putative role of mGSH carriers in cell death and mitochondrial bioenergetics in early passage human RPE cells [81,97]. Pharmacologic inhibition of OGC or DIC caused a significant mGSH depletion and increased cell death in RPE cells [97]. As presented in Figure 4, OGC and DIC carrier protein expression was significantly increased in polarized RPE cells, which mimic features of the native RPE monolayer, including apical localization of Na,K-ATPase and basolateral localization of bestrophin with hexagonal morphology, pigmentation and transepithelial resistance [98]. However, the implication of these findings of elevated mGSH levels in polarized RPE monolayers remains to be evaluated. It was further shown that inhibition of DIC and OGC resulted in mGSH depletion, which significantly decreased mitochondrial respiration, ATP production, and altered ETC protein expression in RPE cells [81]. This was also the case under conditions of silencing OGC siRNA. In addition, chemical inhibition of DIC and OGC caused a tight junction break and a significant drop in TER in polarized human RPE monolayers.

While data support a role for OGC and DIC in importing GSH into the mitochondria, these two carriers together accounted for only an apparent 45–50% of the total GSH uptake in liver mitochondria and 70–80% in kidney mitochondria [99]. A similar observation was also reported in RPE cells in which inhibition of OGC and DIC did not completely block mitochondrial GSH import [97]. This implies the existence of other putative mGSH carriers that are still undescribed. A potential candidate is the uncoupling protein 2 (UCP2), which was recently shown to participate in the transport of mGSH. However, the mechanism remains unclear [100].

The regulation of these mitochondrial transporters is an active area of research. Initial studies suggest that Bcl-2 protein as a potential regulator of mGSH transport by regulating the affinity of OGC for GSH [91]. The coordinated interaction between Bcl-2 and OGC seems to increase the mGSH pool and it has been shown that neuronal cells overexpressing OGC have an increased expression of Bcl-2 protein, an effect that was presumably dependent on the mGSH increase [101]. Therefore, functions attributed to Bcl-2, such as antiapoptotic and potential antioxidant properties, could in part depend on its ability to regulate the mGSH transport and status.

**Figure 5:**
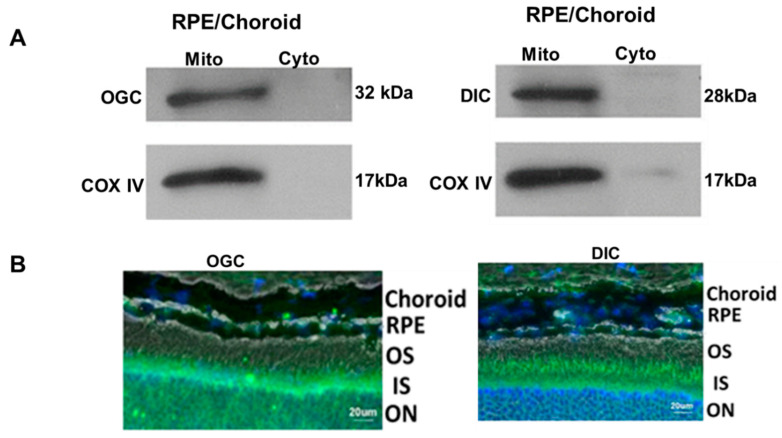
Expression of mGSH carrier proteins in mouse RPE/Choroid (**A**) and whole retina (**B**). Mitochondria was isolated as described earlier [97], and the specificity of expression in mitochondria is illustrated with COX IV as a mitochondria specific marker in A. Immunofluorescence staining of OGC (green) and DIC (green) in retinal layers is shown in B. Mito: mitochondria, Cyto: cytosol, COX IV: cytochrome c oxidase subunit 4, Reproduced from [97] and is licensed under a Creative Commons Attribution-NonCommercial-NoDerivatives 4.0 International License. Blue: nuclear stain, DAPI. OS—outer segment, IS—inner segment, ON—outer nuclear layer.

While most of the characterization of mGSH carrier proteins centered around RPE, it remains to be seen whether these transporters have a role in other retinal cell types. Our recent work reveals that in addition to expression in RPE/choroid, OGC and DIC are also found in the inner nuclear layer of the mouse retina (Figure 5A,B). A recent study of the transcriptional profiles from 453 human donor retinas reported the expression of these two mitochondrial GSH carriers (SLC25A10 and SLC25A11), thereby corroborating the findings in murine retina [102]. Taken together, the presence of OGC and DIC in the retina suggests they may contribute to mitochondrial health in photoreceptors other cells of the neural retina, though additional supportive evidence will be necessary to confirm this postulation [97]. In this context, the few reports of OGC or DIC knockout mice associated with the cancer field begin to shed light on the relative importance of each protein. OGC knockout mice show lethality between embryonic day 10.5 and 14.5, thereby implying that OGC is required for embryonic development [103]. On the other hand, DIC gene knockdown significantly altered NADPH production and cell proliferation, and it was proposed that it could be a novel target in anti-cancer strategies [104]. The phenotype and functional consequences for the retina following genetic ablation of the two GSH carrier proteins are an area for further investigation. In addition, the mechanisms underlying the regulation of OGC and DIC in retinal diseases remain to be explored.

## 8. Mitochondrial GSH and Regulation of Cellular Respiration

**Figure 6:**
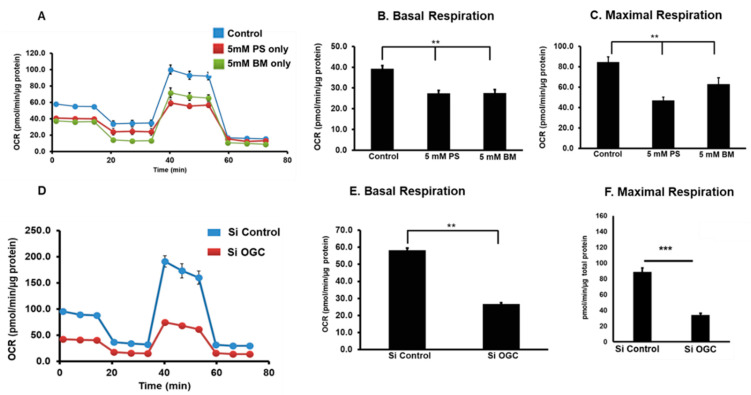
Chemical inhibition of OGC and DIC with PS and BM decreased mitochondrial bioenergetics in RPE (**A**–**C**). Silencing OGC caused a significant decrease in respiratory parameters (**D**–**F**). (Modified from Sreekumar et al. [81] and is licensed under a Creative Commons Attribution-NonCommercial-NoDerivatives 4.0 International License). ** *p* < 0.1 *** *p* < 0.01.

Mitochondria in mammalian cells generate most of the cellular energy via oxidative phosphorylation (OXPHOS). Mitochondria are also involved in several other cellular functions, such as Ca^2+^ homeostasis, heme biosynthesis, nutrient metabolism, steroid hormone biosynthesis, integration of metabolic and signaling pathways for cell death and autophagy [84,105]. Previous work has shown that oxidative stress reduced cellular GSH and altered mitochondrial bioenergetic parameters, such as basal respiration, ATP production, maximum respiration, and proton leak in RPE cells [106]. Recently, the Kannan laboratory selectively inhibited mitochondrial GSH using inhibitors of GSH transporters and studied cellular respiration. Studies were conducted on the effect of mGSH depletion on mitochondrial respiratory parameters following pharmacological inhibition of DIC and OGC or silencing of OGC expression, which reduced mGSH pool by 60–70% [81]. Inhibition of OGC and DIC resulted in a remarkable decrease in basal respiration, maximal O2 consumption, and respiratory reserve capacity, an indicator of cellular bioenergetic resiliency (Figure 6). Dysregulation of ETC proteins was also observed with mitochondrial GSH inhibition. More in-depth studies will be valuable to establish a definitive role of GSH-dependent mitochondrial respiratory functions in RPE.

The decrease in mGSH also modulated known components of the mitochondrial biogenesis machinery [81]. A candid experimental evidence for impaired mitochondrial biogenesis in oxidative stress-induced RPE includes a remarkable reduction in mtDNA copy number and decrease in mitochondrial transcription factor A (mtTFA) expression [106]. mtTFA binds mitochondrial DNA and regulates mitochondrial transcription initiation, mtDNA copy number, packaging of mitochondrial DNA, and mitochondrial biogenesis [107,108]. However, additional in-depth studies are required using multiple biogenesis markers to assess the contribution of mGSH carriers to mitochondrial function.

## 9. Conclusions

Glutathione is an important antioxidant that participates in multiple roles essential for cell survival, such as regulating redox-sensitive proteins and protecting from oxidative and xenobiotic stressors. GSH deficiency renders cells vulnerable to injury and has been associated with several ocular disorders, including AMD, glaucoma, and diabetic retinopathy. GSH is produced in the cytosol and transported into organelles (nucleus, endoplasmic reticulum and mitochondria) via specialized transporters. Two novel GSH transporters recently characterized for the mitochondria were functionally linked to regulation of mitochondrial respiration, thereby providing evidence for the critical role of GSH in maintaining mitochondrial function. Augmentation of the mitochondrial GSH pool through upregulation or activation of these GSH transporters could provide a valuable approach to preventing retinal diseases linked to mitochondrial dysfunction.

## 10. Future Directions

While the SLC25 protein family has been known for many years, the participation of SLC25A10 (DIC) and SLC25A11 (OGC) in the transport of GSH into the mitochondria is a recent finding; therefore, the significance will need further investigation. Some salient points related to this finding that may trigger future research are summarized below.

Only 50–60% of GSH transport to mitochondria is accounted for by the two transporter proteins, suggesting the existence of other undescribed carriers for GSH.

Both OGC and DIC transport multiple substrates and perform other functions. For example, DIC participates in fatty acid synthesis [93] and OGC in insulin secretion [109].

Other upstream Kreb’s intermediate carriers may regulate mGSH and its carriers. Recently, when the mitochondrial pyruvate carrier (MPC), which transports pyruvate from the cytosol into the mitochondrial matrix [110,111], was knocked out, total GSH was found to be 50% lower in the KO retina [112]. Furthermore, retina-specific deletion of MPC1 resulted in progressive retinal degeneration and decline of visual function in photoreceptors [112]. It is to be noted that loss of MPC1 blocks the entry of pyruvate into mitochondria and depletes α-ketoglutarate, a precursor for the synthesis of glutamate, glutamine, and GSH [113].

Analogs and prodrugs are employed for the delivery of GSH because of its poor stability and poor bioavailability [114]. For example, N-acetyl-L-cysteine (a prodrug of L-cysteine) significantly increased GSH levels in RPE in vitro [59]. It would be of interest to study whether this prodrug will be applicable to prevent retinal degeneration in animal models, specifically those that exhibit elevated oxidative stress in the RPE monolayer (for example, in *Sod2* knockout mice [115]). Whether the prodrug selectively upregulates mGSH or its carriers will be worthy of investigation.

Mitochondrial-targeting agents based on peptides possess remarkable advantages and can augment GSH in GSH-deficient conditions. For example, a dendrimer-drug construct (TPP-D-NAC) with triphenyl phosphonium (TPP) for mitochondrial targeting of NAC was shown recently to offer protection in a brain injury model [116]. Such an approach can also be utilized for upregulating mGSH with endogenous and mitochondrial-derived peptides [49,81,117]. For example, we have shown that incubation of RPE cells with a mitochondria-derived peptide, humanin, significantly upregulated mGSH [117].

Finally, studies on how changes in the mGSH status will regulate key events related to mitochondrial dysfunction, such as autophagy/mitophagy and inflammation, will be fruitful to pursue.

## Figures and Tables

**Table 1:** Regulation of GSH in retinal diseases.

Pathology	GSH Levels		Model (s) Studied	Ref
Wolfram syndrome 1 (WS)	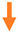	Total GSH	Wfs1−/− mice	[44]
Wolfram syndrome	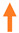	GSSG/GSH and NAD(+)/NADH ratios	Miner1(−/−) mice	[45]
Age-related macular degeneration (AMD)	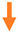	Plasma Total GSH	Human AMD patients	[46]
Exudative AMD	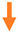	Plasma Total GSH	Human ex-AMD patients	[47]
Exudative AMD	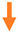	Plasma Total GSH	Human ex-AMD patients	[48]
AMD	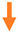	Mitochondrial GSH	hRPE cells and RPE from alpha Cry KO mice	[49]
AMD	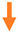	Cellular GSH	ARPE-19 cells treated with BSO or erastin	[50]
AMD	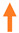	Oxidized glutathione (GSSG) in plasma	Human early AMD patients	[24]
Dry AMD	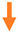	Total GSH	RPE cells from AMD donors subjected to H2O2	[51]
AMD	N-acetylcysteine (precursor for GSH) improved maximal respiration and ATP production		RPE cells from AMD donors	[52]
AMD	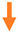	Cellular GSH, ATP production, and basal respiration	RPE from AMD donors and non-AMD with and without H2O2 treatment	[21]
AMD	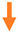	GSH:GSSG ratio	ARPE19 cells subjected to H2O2 exposure	[53]
AMD	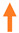	Ocular GSH	ARPE 19 incubated with and rats fed N-acetyl-L-cysteine ethyl ester or N-acetyl-L-cysteine	[54]
AMD	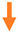	GSH	LAMP2-silenced ARPE-19 cells.	[55]
AMD	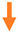	GSH	ARPE19 cells stressed with tert-Butyl hydroperoxide	[56]
AMD	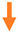	GSH	ARPE-19/primary human RPE cells were exposed to cigarette smoke extract or hydroquinone	[57]
AMD	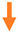	GSH	ARPE-19 cells treated with H2O2	[58]
AMD	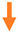	Cellular GSH	ARPE-19 cells treated with hydroquinone	[59]
AMD	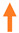	Cellular and mitoGSH	ARPE-19 treated with NAC prodrugs	[59]
AMD	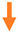	GSH	ARPE-19 cells treated with H2O2	[60]
Diabetic retinopathy	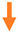	MitoGSH	Streptozotocin (STZ) diabetic mice	[61]
Diabetic retinopathy	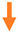	Retinal GSH	STZ-injected Nrf2−/− mice	[62]
Diabetic retinopathy	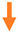	GSH	STZ rats	[63]
Glaucoma	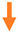	Plasma GSH levels	Human glaucoma patients	[64]
Glaucoma	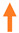	GSSG levels	Human peripheral blood mononuclear cells	[65]
Glaucoma	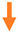	Plasma GSH	Human blood	[22]
Retinitis pigmentosa	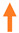	Increased GSH	rd1(+/+) and rd10(+/+) mice treated with NAC	[66]
Retinitis pigmentosa	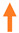	GSSG level	rd10 mouse model	[67]

## Abbreviations

ABC transporter	ATP-Binding Cassette transporter
AMD	Age-related macular degeneration
ATP	Adenosine triphosphate
BM	Butylmalonate
Cu/Zn-SOD	Cu/Zn-superoxide dismutase1
DIC	Dicarboxylate carrier
EMT	Epithelial-mesenchymal transition
ER	Endoplasmic reticulum
ETC	electron transport chain
G6PD	Gucose-6-phosphate dehydrogenase
GCL	Glutamate–cysteine ligase
GCLC	Glutamate–cysteine ligase catalytic subunit
GCLM	Glutamate–cysteine ligase modifier subunit
GPx	Glutathione peroxidase
GR	Glutathione reductase
GS	Glutathione synthetase
GSH S-transferases	Glutathione S-transferases
GSH	Glutathione
GSH-Px	Glutathione peroxidase
GSHR	Glutathione reductase
GSSG	Glutathione disulfide
IMM	Inner mitochondrial membrane
IMS	Intermembrane space
mGSH	mitochondrial GSH
*MnSOD*	Manganese superoxide dismutase
MPC	Mitochondrial pyruvate carrier
MRP	Multidrug resistance-associated protein
mtDNA	Mitochondrial DNA
mtTFA	Mitochondrial transcription factor A
*NAC*	N-acetyl cysteine
NADPH	Nicotinamide adenine dinucleotide phosphate
OGC	2-oxoglutarate carrier
OMM	Outer mitochondrial membrane
OXPHOS	Oxidative phosphorylation
PS	Phenyl succinate
6PGD	6-phosphogluconate dehydrogenase
RPE	Retinal pigment epithelium
SLC25A10	Solute Carrier Family 25 Member 10
SLC25A11	Solute Carrier Family 25 Member 11
TER	Transepithelial resistance
UCP2	Uncoupling protein 2
